# Effect of palladium(II) complexes on NorA efflux pump inhibition and resensitization of fluoroquinolone-resistant *Staphylococcus aureus*: *in vitro* and *in silico* approach

**DOI:** 10.3389/fcimb.2023.1340135

**Published:** 2024-01-15

**Authors:** Rajaramon Shobana, Jaffer Hussain Thahirunnisa, Selvam Sivaprakash, Arlin Jose Amali, Adline Princy Solomon, Devarajan Suresh

**Affiliations:** ^1^ Quorum Sensing Laboratory, Centre for Research in Infectious Diseases (CRID), School of Chemical and Biotechnology, SASTRA Deemed to be University, Thanjavur, India; ^2^ Organometallics and Catalysis Laboratory, Department of Chemistry, School of Chemical and Biotechnology, SASTRA Deemed University, Thanjavur, Tamil Nadu, India

**Keywords:** *Staphylococcus aureus*, palladium, metal complex, NORA, efflux pump, biofilm, drug resistance

## Abstract

*Staphylococcus aureus* leads to diverse infections, and their treatment relies on the use of antibiotics. Nevertheless, the rise of antibiotic resistance poses an escalating challenge and various mechanisms contribute to antibiotic resistance, including modifications to drug targets, enzymatic deactivation of drugs, and increased efflux of antibiotics. Hence, the quest for innovative antimicrobial solutions has intensified in the face of escalating antibiotic resistance and the looming threat of superbugs. The NorA protein of *S. aureus*, classified as an efflux pump within the major facilitator superfamily, when overexpressed, extrudes various substances, including fluoroquinolones (such as ciprofloxacin) and quaternary ammonium. Addressing this, the unexplored realm of inorganic and organometallic compounds in medicinal chemistry holds promise. Notably, the study focused on investigating two different series of palladium-based metal complexes consisting of QSL_P^A^ and QSL_P^B^ ligands to identify a potent NorA efflux pump inhibitor that can restore the susceptibility to fluoroquinolone antibiotics. QSL_Pd^5A^ was identified as a potent efflux pump inhibitor from the real-time efflux assay. QSL_Pd^5A^ also resensitized SA1199B to ciprofloxacin at a low concentration of 0.125 µg/mL without elucidating cytotoxicity on the NRK-62E cell line. The *in vitro* findings were substantiated by docking results, indicating favorable interactions between QSL_Pd^5A^ and the NorA efflux pump.

## 1 Introduction

The pursuit of novel antimicrobial compounds in the era of antibiotic resistance has garnered significant attention due to the relentless progression of superbugs, posing a global threat to human health (Álvarez-Martínez et al., 2020). The escalation of multidrug resistance (MDR) and antibiotic evasion is surpassing the pace at which the scientific community is developing new antibiotics. The World Health Organization (WHO) projects that antibiotic-resistant bacteria’s swift evolution and dissemination will precipitate a global medical crisis. Furthermore, the Centers for Disease Control and Prevention reports an annual occurrence of approximately 2.8 million infections and 35,900 fatalities attributed to antibiotic resistance (Rajaramon et al., 2023).

Following several decades, in the year 2019, the antibiotic drug development landscape appeared to rebound from its scarcity, marked by the presence of approximately 42 antibiotic drug candidates undergoing clinical trials (Nasiri Sovari and Zobi, 2020). Nonetheless, a mere 25% of the primary compounds fall within novel structural categories, with the remainder being derivations of pre-existing antibiotics. Notably, all scrutinized lead compounds within pre-clinical and clinical trials are exclusively organic, which poses the risk of organisms swiftly developing multiple resistance mechanisms (Frei, 2020). Hence, the desperate need for the development of not only new antimicrobial agents but also with a new, diverse structural class is required to tackle the emergence of antimicrobial resistance.

Inorganic compounds, organometallic compounds, and metal complexes in the medicinal chemistry field remain unexplored drug sources (Frei et al., 2023). Although it has been employed since ancient days, its application was confined to material synthesis or as a catalyst (Sodhi, 2019). It was not preferred as an antibiotic as it was often associated with toxicity. Over the last decade, a light dawned on the “metallodrugs” after the discovery and regulatory approval for various ailments such as Syphilus (Salvarasan/Arsphenamine) and cancer (cisplatin) (Lloyd et al., 2005; González‐Ballesteros et al., 2022). The activity of the transition metal complexes is attributed to their three-dimensional geometry, coordination properties, and various oxidation states (Zhang and Sadler, 2017). Their mode of action includes inhibition of enzymes, depolarization of mitochondria, reactive oxygen species generation, metal–ligand interchange, and redox reaction (de Sousa et al., 2020). In addition, the electropositive nature of transition metals allows them to solubilize in biological fluids with no hindrance in bioavailability. The positive ions released tend to bind to protein and DNA base pairs, forming an inner-sphere complex (Sharma et al., 2022). However, achieving the aforementioned mechanisms with organic compounds can be quite challenging due to their inherent structural limitations.

In recent times, there has been a rise in the study and advancement of metal complexes, although the number of organic compounds still surpasses it due to concerns about toxicity. However, it is possible to reduce the level of toxicity by modifying the ligand, which can help minimize its effects on the biological components of humans (Egorova and Ananikov, 2017). In addition, the kinetic properties and lipophilicity of transition metal complexes and their interaction with biomolecules can also be altered by modifying ligands (Claudel et al., 2020). Considering various factors, the three-dimensional space can be exploited by virtually designing various metal–ligand combinations to achieve appropriate physical, chemical, and electronic properties to overcome antibiotic resistance exhibited due to biofilm formation, overexpression of efflux pumps (EPs), and/or degradation of antibiotics.

*S. aureus*, an opportunistic, biofilm-forming, and high-priority pathogen, is known to cause several uncomplicated skin infections and is likely to contribute to severe, invasive infections worldwide annually (Cheung et al., 2021). According to WHO, *S. aureus* is a high-priority pathogen and is among the major five infections causing high mortality and morbidity rates every year worldwide (WHO, 2017). In the year 2019, *S. aureus* emerged as a predominant pathogen in numerous countries and was known to be linked to over 1 million fatalities and 34 million years of life lost worldwide (Ikuta et al., 2022). Treatment for these infections is increasingly more challenging due to the emergence of MDRs like methicillin-resistant *Staphylococcus aureus* (MRSA) and vancomycin-resistant *Staphylococcus aureus* (VRSA) (Chen and Huang, 2014; Piechota et al., 2018). EPs, being a first line of defense, are one of the critical factors leading to the multi-drug resistance of pathogens (Costa et al., 2013a). The ability of multi-drug EPs to evict noxious compounds and antibiotics renders the drug less effective. The EP present in *Staphylococcus aureus*, namely, NorA, belonging to the major facilitator superfamily is one of the most extensively studied EPs (Alav et al., 2018). Strategies for developing EP inhibitors (EPIs) can be classified according to their mode of action or compound origin (Sharma et al., 2019). The modes of action include energy-dissipating inhibitors and direct binding inhibitors. An example of widely used energy-dissipating inhibitor is carbonyl cyanide-m chlorophenylhydrazone and direct binding inhibitor is verapamil (Sharma et al., 2019). EPIs can also originate for from various sources, such as plant extracts (e.g., piperine and reserpine), synthetic EPIs, or microbial derivatives. EPIs can originate from various sources, such as plant extracts (e.g., piperine and reserpine), synthetic EPIs, or microbial derivatives. To date, bounteous transition metals/metal complexes under groups 6–12 (Ag, Au, Zn, Cu, Co, Pt, Pd, Ru, and Ga) have been identified as anti-bacterial and anti-biofilm agents (Nasiri Sovari and Zobi, 2020). In this context, an interdisciplinary approach encompassing medicinal chemistry, microbiology, and bioinformatics was utilized to validate a series of innovative palladium-based metal complexes. The objective was to identify a novel EPI capable of re-sensitizing the NorA-overexpressing strain to fluoroquinolone antibiotics.

## 2 Materials and methods

### 2.1 Chemicals and reagents utilized

Microbiological culture media and chemical reagents were procured from HiMedia, Mumbai. Antibiotics were obtained from SRL Fine Chemicals. The LIVE/DEAD BacLight Bacterial Viability Kit (L-7012) was sourced from Thermo Fisher Scientific in the United States.

### 2.2 Bacterial strains and conditions

Bacterial strains SA1199 (wild type) and SA1199B (overexpressing *norA*) were obtained from BEI Resources, NIAID, NIH, USA. The strains were retrieved from glycerol stock (−80°C) and regularly sub-cultured, maintaining them on Tryptic Soy Agar (TSA). Confirmation of SA1199B as a fluoroquinolone-resistant strain due to NorA overexpression was carried out through antimicrobial susceptibility testing using the broth microdilution method as per Clinical and Laboratory Standards Institute guidelines (Limbago, 2001).

### 2.3 Source of palladium metal complexes

The ligands (QSL_P^A^ and QSL_P^B^) and their palladium(II) metal complexes (QSL_Pd^1A^ to QSL_Pd^6A^ and QSL_Pd^1B^ to QSL_Pd^6B^) were developed through collaboration with Dr. D. Suresh, Assistant Professor of Research at the School of Chemical and Biotechnology, SASTRA Deemed University.

### 2.4 Minimum inhibitory concentration of synthesized compounds

The minimum inhibitory concentration (MIC) for the synthesized ligands and their palladium(II) metal complexes (“A” and “B” series) was determined by following CLSI guidelines with slight modifications (Limbago, 2001). A stock solution containing all the synthesized compounds was meticulously prepared in dimethyl sulphoxide (DMSO), achieving a concentration of 22 mg/mL. The strains SA1199 and SA1199B were cultured overnight in tryptic soy broth (TSB). The culture was diluted 1:100 in physiological saline, and the bacterial suspensions were adjusted to 0.5 Mc Farland standard. Employing a 96-well microtiter plate, the adjusted inoculum was added to the wells containing two-fold serial dilutions of the compound covering a concentration range from 600 µg/mL to 1.71 µg/mL in Cation-adjusted Mueller Hinton broth (CAMHB). Following a 24-h incubation at 37°C, cell viability was measured at OD_595_, and the extent of inhibition was quantified using the following formula:


$$\frac{\left(\text{OD of Control }-\text{ OD of Test}\right)\;*\;100}{\left(\text{OD of Control }-\text{ OD of Blank}\right)}$$

### 2.5 EtBr cartwheel assay

For qualitative assessment of EP inhibition induced by the synthesized ligands and palladium(II) complexes, a cartwheel assay was conducted following the protocol outlined by Martins et al., with minor adaptations (Martins et al., 2013). EPs use ethidium bromide (EtBr) as the substrate to extrude, hence used to detect the efflux activity by EtBr cartwheel assay. Only compounds from the “A” series were selected on the basis of MIC outcomes. A consistent concentration of EtBr at 1 µg/mL and compounds at 0.5× MIC were incorporated into molten TSA at 50°C. The resulting agar mixture was poured into petri plates and allowed to solidify. Cultures of bacterial strains SA1199 and SA1199B were adjusted to a density of 0.5 McFarland units (1.5 × 10^8^ Colony forming units (CFU)/mL) and were systematically swabbed onto the plates in a cartwheel pattern, with triplicate samples. Following incubation for 24 h at 37°C, fluorescence was visualized using a UV-transilluminator.

### 2.6 Real-time efflux assay

For a quantitative assessment of EP inhibition induced by the synthesized palladium(II) complexes and ligands, a real-time efflux assay was conducted following the protocol outlined by Costa et al., with minor adaptations (Costa et al., 2013b). The overnight culture of SA1199B was subjected to centrifugation, washed twice with PBS supplemented with 0.4% glucose, and subsequently re-suspended in an equivalent volume of media and the turbidity was adjusted in the uptake buffer, followed by the addition of EtBr (10 µg/mL). The cells were incubated at 37°C for 1 h to facilitate EtBr uptake. After centrifugation at 12,000 rpm for 5 min, the cells underwent two washes with phosphate buffered saline (PBS) (0.4% glucose). Four palladium(II) complexes demonstrating elevated fluorescence in the EtBr cartwheel assay, one ligand(QSL_P^A^) and verapamil as control at 0.5× MIC were each introduced into a 96-well black tissue culture plate. Subsequent readings were taken at 5-min intervals over a span of 30 min using the Biotek Synergy H1 multimode reader, with excitation at 530 nm and emission at 600 nm. The compound displaying stronger EP inhibition in the real-time efflux assay was selected for subsequent combinatorial investigations.

### 2.7 Antibiofilm assay

The effectiveness of QSL_Pd^5A^ in inhibiting biofilm formation was assessed against SA1199B using the crystal violet assay. The overnight culture of SA1199B was suitably diluted, following the procedure detailed in the MIC determination, and introduced into a 96-well plate containing sub-inhibitory concentrations of QSL_Pd^5A^ (ranging from 9.38 µg/mL to 0.29 µg/mL in two-fold serial dilutions). The culture medium employed was TSB supplemented with 1% NaCl and 1% glucose. After 24 h of incubation at 37°C, the planktonic cells were measured for their optical density at OD_595_. The next steps involved gently tapping to remove the planktonic cells, followed by two PBS rinses, and air-drying for 5–10 min. Subsequently, a 0.2% crystal violet (120 µL) was added and incubated for 15 min to achieve cellular staining. After removing the excess stain by washing it with water, the plate was air-dried and eluted with 33% acetic acid. The absorbance was quantified at 595 nm (Vasudevan et al., 2020). This meticulous process was conducted in triplicates to ensure robust results.

### 2.8 Microscopic analysis for biofilm inhibition

Fluorescence microscopy imaging was employed to visually investigate the biofilm inhibition potential of QSL_Pd^5A^. In a concise description of the method, the inoculum was cultured in a six-well plate with 2 mL of TSB supplemented with 1% glucose and 1% NaCl. Sterile coverslips were positioned, both in the presence and absence of the compound. Different concentrations, including 0.5×, 0.25×, and 0.125× the MIC of QSL_Pd^5A^, as well as 0.5× MIC of QSL_P^A^ and 0.5× MIC of verapamil (used as a positive control), were introduced into appropriate wells. Untreated wells were retained as controls. After 24 h of incubation at 37°C, non-adherent planktonic cells were carefully removed, and the coverslips were gently washed twice with PBS. Utilizing the Bac Light Bacterial Viability Kit (L7012), coverslips were stained as per the manufacturer’s instructions using propidium iodide and Syto9 dyes, followed by a 10-min dark incubation. After eliminating excess stains and conducting two PBS washes, the coverslips were meticulously mounted onto glass slides. Subsequently, fluorescence excitation images were adeptly captured at a ×20 magnification using the Nikon Eclipse Ts2 microscope.

### 2.9 Checkerboard assay

The checkerboard assay was performed to assess the potentiating activity of QSL_Pd^5A^ with ciprofloxacin, following the guidelines outlined by the CLSI (Limbago, 2001). Briefly, QSL_Pd^5A^ starting from its sub-inhibitory concentration of 9.38 µg/mL was two-fold serially diluted in the wells containing 100 µL of CAMHB. This was combined with an addition of ciprofloxacin concentrations spanning from 4 µg/mL to 0.125 µg/mL. The individual concentrations of both compounds were also plated to serve as controls and references for the synergistic effects observed. Following the approach described in the MIC experiment, the overnight culture of SA1199B was suitably diluted and inoculated in 96-well plates (Thamilselvan et al., 2021). The plates were incubated for 24 h at 37°C, the optical density was measured at 595 nm and, subsequently, the percentage of inhibition was calculated. The obtained data were analyzed using SynergyFinder Plus, utilizing the bliss independence model. This model calculates the expected effect based on the product of individual effects, represented by the equation Ei = EA * EB.

### 2.10 *In vitro* time-kill kinetics

The time-kill efficacy of the combination of ciprofloxacin (CIP) with QSL_Pd^5A^ was investigated to gain insights into CIP’s impact on SA1199B in the presence of QSL_Pd^5A^ over 24 h. The experimental protocol closely mirrored that of the checkerboard assay. Succinctly, CIP at a concentration of 0.125 µg/mL was combined with QSL_Pd^5A^ at a concentration of 4.69 µg/mL, as determined from the outcomes of the checkerboard assay. These concentrations were added in volumes of 100 µL to each well of a 96-well plate, along with 10 µL of the bacterial culture. The untreated bacterial culture was treated as a negative control. At intervals of every 2 h, readings were taken at OD_595_ from the 0th hour through the 10th hour, culminating in a final reading at the 24th hour.

### 2.11 Toxicity evaluation

To determine the cytotoxic effect of QSL_Pd^5A^, an 3-(4,5-Dimethylthiazol-2 yl)-2,5-Diphenyltetrazolium Bromide (MTT) assay was performed (Balamurugan et al., 2017). Eagle’s Minimum Essential Medium supplemented with non-essential amino acids, 10% fetal bovine serum, and penicillin-streptomycin (100 U/mL to 100 µg/mL) was used as culture medium. In brief, 200 µL of viable human kidney epithelial cells (NRK-62E) in a concentration of 1.5 × 10^4^ cells/mL were seeded in 96-well tissue culture plates. At 80% confluence, the varying concentrations of QSL_Pd^5A^ (1×, 10×, and 100× MIC) were added to each well. The plate was incubated at 37°C for 24 h with 5% CO_2_. Furthermore, 20 µL of MTT solution (5 mg/mL) was added to each well and incubated for 3 h. Following the incubation period, 200 µL of DMSO was added to the wells, and optical density (OD) was taken at 570 nm. The percentage cell viability was calculated with reference to the untreated control cells using the below-given formula:


$$\text{Percentage cell viability }=\frac{(\text{OD of Drug }-\text{ Treated sample }-\text{ OD of Blank})*100}{\left(\mathrm{OD of Control}-\mathrm{OD of Blank}\right)}$$

### 2.12 Docking studies

Docking simulations were conducted between the NorA EP (PDB ID: 7LO7) and QSL_Pd^5A^ using the Autodock 4.2 suite. The NorA protein structure model, acquired from a previous study conducted by our lab using the I-TASSER (Iterative Threading ASSEmbly Refinement) server (https://zhanglab.dcmb.med.umich.edu/I-TASSER/) (Thamilselvan et al., 2021), was employed. The protein preparation phase involved reading the NorA protein protein data bank (PDB) file, addressing missing atoms and adding polar hydrogen atoms. Kollman charges were subsequently incorporated into the protein, and the resulting file was saved as a.pdbqt file. During ligand preparation, the ligand molecule’s 2D structure was drawn using MarvinSketch (https://chemaxon.com/products/marvin) and saved as a 3D coordinate.pdb file. The ligand, QSL_Pd^5A^, underwent optimization and energy minimization via Avogadro 1.2, employing the UFF force field and a conjugate gradient approach with 2,000 steps. The optimized ligand configuration was integrated into Autodock 4.2, followed by the addition of missing atoms and polar hydrogen. Gasteiger charges were applied to the ligand, and the result was saved as a.pdbqt file. Subsequent steps encompassed grid box generation, centered on the ligand binding site featuring residues Ile23, Pro24, Leu26, Pro277, Phe47, Arg98, Val144, Tyr225, and Gly348. The parameters for palladium(II) metal were sourced from the Autodock website. Docking procedures utilized the genetic algorithm search method with 50 conformations and 300 steps, yielding an output file derived from the Lamarkian genetic algorithm. The output file was scrutinized to identify the most optimal docking complex, as indicated by lower binding energy. The resulting complex was visualized using PyMOL, with interaction visualization facilitated by LigPlus Version V.2.2.8. Similar methodologies were applied to perform docking simulations for the positive control, verapamil, and the negative control ligand, QSL_P^A^, aiming to comparatively assess the binding affinity of QSL_Pd^5A^.

### 2.13 Statistical analysis

GraphPad Prism software version 8.0.2 (GraphPad Software Inc., San Diego, CA, United States) was employed for conducting the statistical analysis. To assess significance, a Student’s t-test was executed, with the minimum level of significance established at p ≤ 0.05. All assays were performed in triplicate, and the outcomes were presented as mean ± standard deviation (SD).

## 3 Result

### 3.1 Synthesis of palladium(II) metal complexes

**Figure 1:**
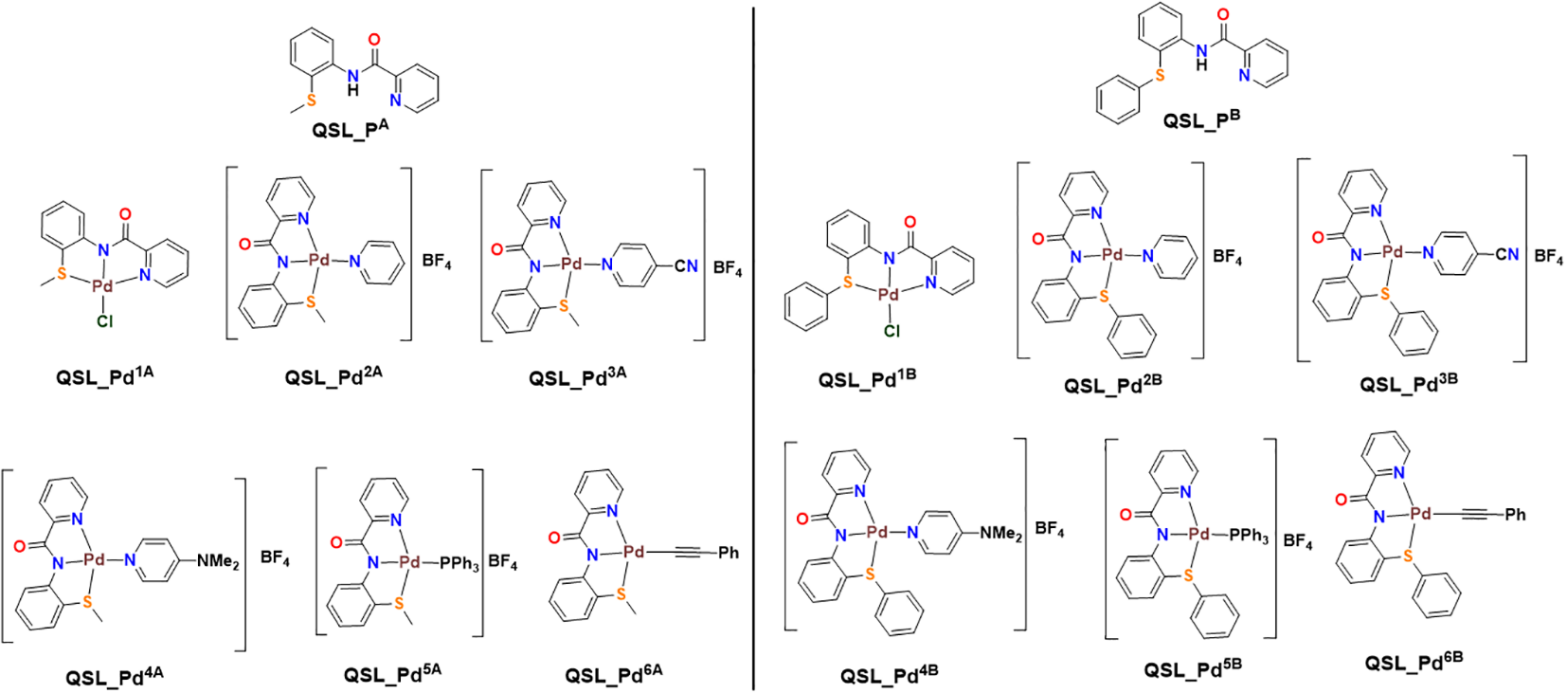
Structure of ligand (QSL_P^A^ and QSL_P^B^) and palladium(II) metal complexes. Left: QSL_Pd^1A^ to QSL_Pd^6A^ (“A” series). Right: QSL_Pd^1B^ to QSL_Pd^6B^ (“B” series).

The ligands, QSL_P^A^ (“A” series) and QSL_P^B^ (“B” series), and their palladium(II) complexes, QSL_Pd^1A^ to QSL_Pd^6A^ and QSL_Pd^1B^ to QSL_Pd^6B^, were synthesized through coupling reactions of commercially available α–picolinic acid with 2-(methylthio)aniline (for “A” series) or 2-(phenylthio)aniline (for “B” series) (**Figure 1**). The newly synthesized ligands and their complexes were characterized through ^1^H and ^13^C{1H} NMR spectroscopic techniques and few of them were confirmed through single-crystal X-ray diffraction studies. The details of the synthesis and characterization will be disclosed elsewhere.

### 3.2 Minimum inhibitory concentration

The ligand of both “A” series (QSL_P^A^) and “B” series (QSL_P^B^) of palladium(II) complexes had no inhibition activity against SA1199 and SA1199B. The palladium(II) ion-centered complexes, “A” series (QSL_Pd^1A^ - QSL_Pd^6A^) and “B” series (QSL_Pd^1B^ - QSL_Pd^6B^) exhibited minimum inhibitory concentration (MIC)_50_ across a varying concentration of 600 µg/mL to 1.71 µg/mL in both the strains and dose–response graph was plotted to mark the MIC_50_ (**Figures S1, S2**). A heat map for the MIC was plotted for both strains to correlate the concentrations (**Figure S3**). The palladium(II) complexes belonging to the A series were identified to display a lower MIC_50_ than the “B” series (**Tables 1**, **2**). Thus, the “A” series of palladium(II) metal complexes were considered for further assays.

**Table 1:** Minimum inhibitory concentrations (MICs) of palladium(II) complexes.

S.No	Compound	Inhibitory concentration (IC)_50_ (µg/mL)	
		SA1199	SA1199B
1	QSL_P^A^	–	–
2	QSL_Pd^1A^	24.7	26.24
3	QSL_Pd^2A^	7.28	13.97
4	QSL_Pd^3A^	9.94	6.66
5	QSL_Pd^4A^	10.48	10.11
6	QSL_Pd^5A^	10.2	18.75
7	QSL_Pd^6A^	17.58	9.14

“A” series against SA1199 and SA1199B strains (n = 3).

**Table 2:** Minimum inhibitory concentrations (MICs) of palladium(II) complexes.

S.No	Compound	IC_50_ (µg/mL)	
		SA1199	SA1199B
1	QSL_P^B^	–	–
2	QSL_Pd^1B^	39.44	67.84
3	QSL_Pd^2B^	41.74	59.51
4	QSL_Pd^3B^	14.35	29.86
5	QSL_Pd^4B^	42.28	31.71
6	QSL_Pd^5B^	9.09	20.27
7	QSL_Pd^6B^	27.09	9.87

“B” series against SA1199 and SA1199B Strains (n = 3).

### 3.3 EtBr-Cart wheel assay

**Figure 2:**
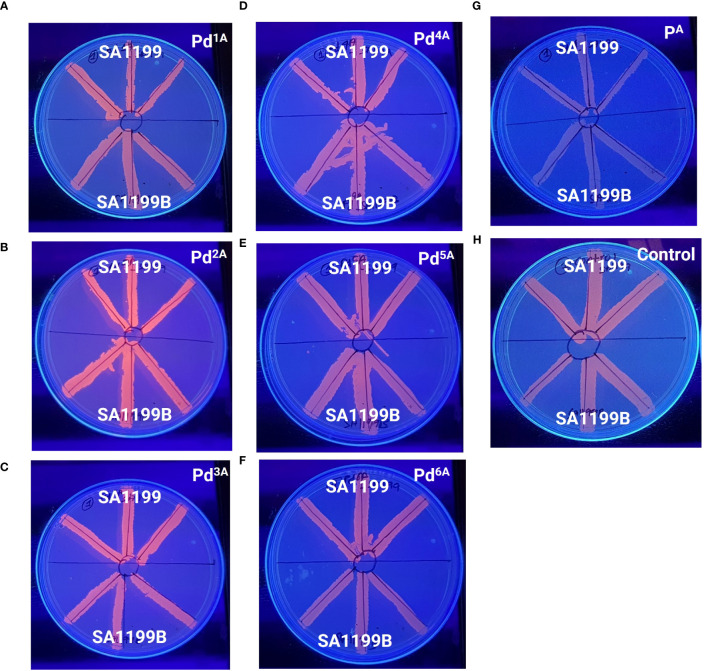
Determination of efflux pump inhibitory activity based on fluorescence emission of palladium(II) complexes (“A” series) at 0.5× MIC **(A–F)** and the ligand **(G)** with respect to the untreated control **(H)** in the presence of EtBr (1 µg/mL) by a cartwheel assay. The plates were visualized using a UV-transilluminator after 24 h.

The MDR EP in *S. aureus* is a major contributor to the antibiotic resistance mechanism. NorA-overexpressing strains are known to be less susceptible to antibiotics, as they expel various classes of antibiotics, notably fluoroquinolones. The Pd “A” series was qualitatively evaluated for their EP inhibitory activity, and the basal level fluorescence emitted by the control was measured and compared with the Pd “A”-series complexes (QSL_Pd^1A^ to QSL_Pd^6A^) and their ligand (QSL_P^A^). The ligand and the untreated plates were taken as a negative control. High fluorescence emission indicates that the compound inhibits the EP and accumulates EtBr inside the cell and vice versa. From **Figure 2**, the results were categorized into three groups on the basis of the fluorescence intensity. The first group consisted of only one compound QSL_Pd^2A^ that displayed high fluorescence emission and, hence, had a negative efflux. The second group and third group consisted of QSL_Pd^1A^, QSL_Pd^3A^, and QSL_Pd^5A^ and of QSL_Pd^4A^ and QSL_Pd^6A^, respectively (**Table S1**).

### 3.4 Real-Time efflux assay

**Figure 3:**
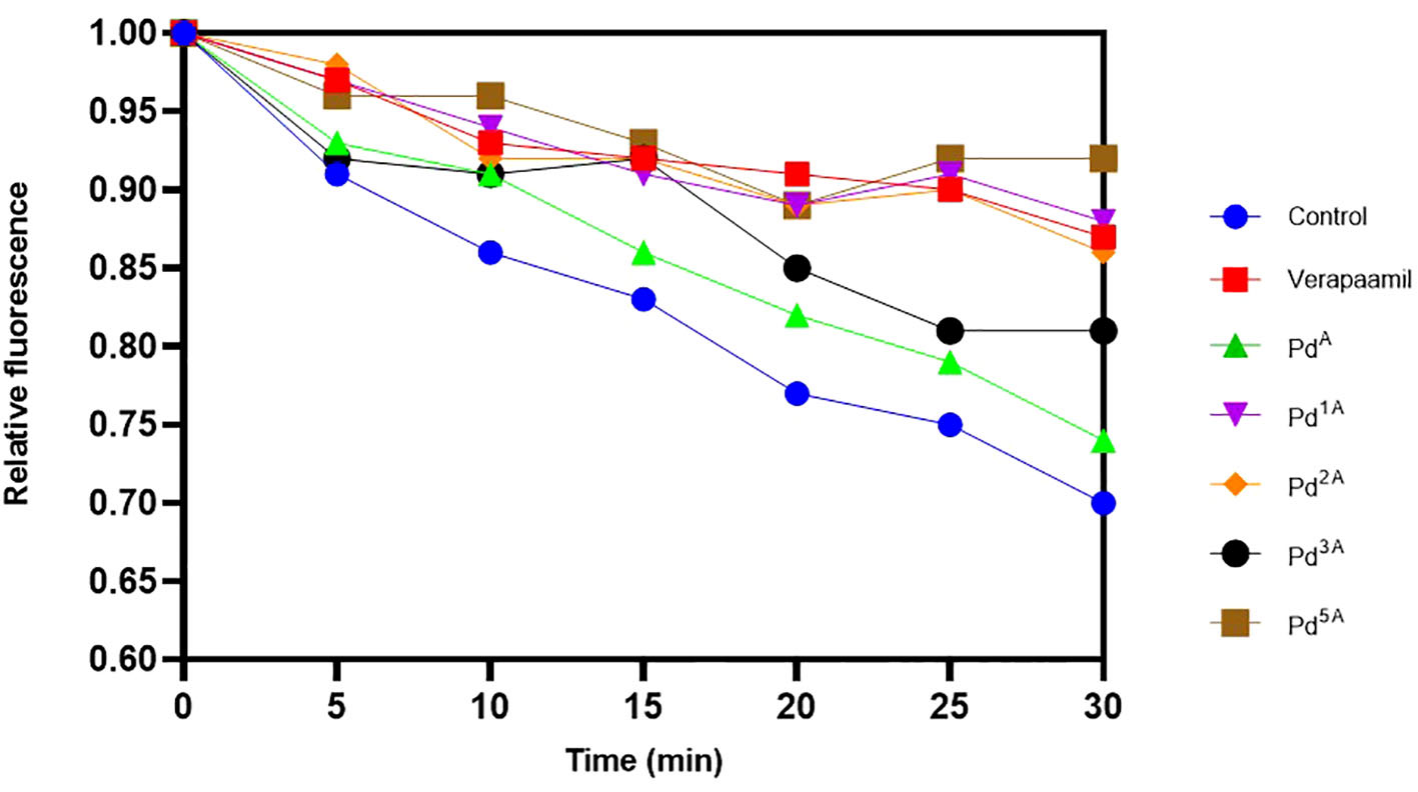
Evaluation of the effect of palladium(II) complexes on NorA efflux pump effluxing activity and the accumulation of ethidium bromide in SA1199B. Positive control, verapamil; and negative control, untreated cells.

The quantitative EP inhibition activity of palladium(II) complexes that exhibited high and intermediate fluorescence (QSL_Pd^2A^, QSL_Pd^1A^, QSL_Pd^3A^, and QSL_Pd^5A^) in EtBr cartwheel assay against SA1199B was tested. A time-dependent decrease in the fluorescence was noted in control (untreated) and cells treated with ligand (QSL_P^A^) due to the action NorA-mediated efflux of EtBr. On the contrary, the cells treated with the complex exhibited fluorescence retention. The palladium(II) complex QSL_Pd^5A^ and QSL_Pd^1A^ had higher relative fluorescence than the verapamil, a known EPI, which indicates that the inhibition of EP activity is more by QSL_Pd^5A^ and QSL_Pd^1A^ (**Figure 3**). The cells treated with QSL_Pd^5A^ and QSL_Pd^1A^ exhibited 1.05-fold and 1.01-fold increase in fluorescence compared to verapamil. Thus, QSL_Pd^5A^ was identified as a potent NorA EPI and was taken for further studies.

### 3.5 Antibiofilm assay and visualization

**Figure 4:**
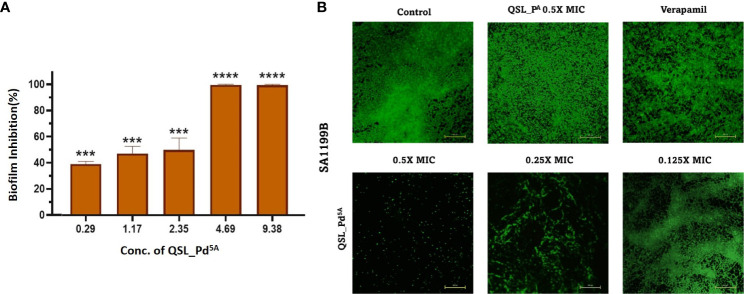
Effect of QSL_Pd^5A^ on biofilm formation. **(A)** The inhibition of biofilm with varying concentrations of QSL_Pd^5A^ assessed using crystal violet assay (n = 3, ***p< 0.0001, **** p< 0.0001 compared with control). **(B)** Overlayed fluorescence (green, live cells; and red, dead cells) images of SA1199B stained with SYTO9/PI after different treatments to visualize biofilm. Control as untreated; verapamil and QSL_P^A^ as positive control; 0.5× MIC, 0.25× MIC, and 0.125× MIC of QSL_Pd^5A^ as treated cells.

A significant reason for chronic and hospital-acquired infections by *S. aureus* is attributed to biofilm formation. Hence, we examined the effect of QSL_Pd^5A^ in their sub-MIC (9.38 µg/mL to 0.29 µg/mL) on biofilm using crystal violet assay. A dose-dependent inhibition was obtained with 9.38 µg/mL and 4.69 µg/mL exhibiting highly significant biofilm reduction (∼99%) compared to the control (**Figure 4A**). The Live/Dead staining and fluorescence microscopy images visually substantiated the biofilm assay results. There was a significant biofilm reduction (>90%) with the treatment of QSL_Pd^5A^ at 0.5× and 0.25× MIC concentrations. A strong biofilm network was not visually observed in 0.125× MIC, although the percentage of biofilm inhibited was less. The ligand QSL_P^A^ had no effect on the inhibition of biofilm (**Figure 4B**).

### 3.6 Checkerboard assay

**Figure 5:**
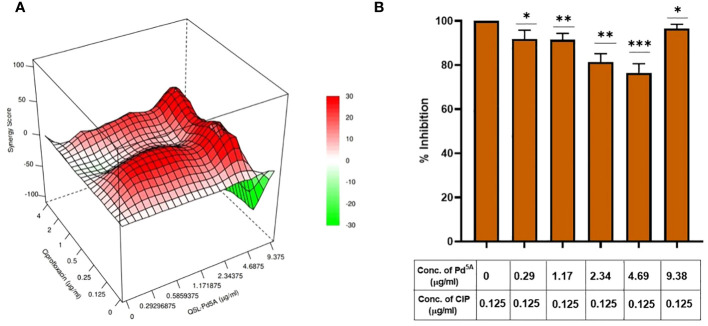
Effect of QSL_Pd^5A^ in enhancing the bactericidal potential of ciprofloxacin. **(A)** Synergy plot of SA1199B at varying concentrations of QSL_Pd^5A^ and ciprofloxacin. **(B)** Potentiation activity of QSL_Pd^5A^ was obtained at the minimum concentration of 4.69 µg/mL and constant concentration of ciprofloxacin at 0.125 µg/mL for SA 1199B.

A checkerboard assay was performed to evaluate the effect of CIP in the presence of QSL_Pd^5A^, and the bliss independence model was used to assess the combinatorial effect. According to the model, the bliss score > 0 is synergistic, bliss score < 0 is an antagonistic effect, and bliss score = 0 is called an additive effect. The combinatorial action with varying concentrations of QSL_Pd^5A^ (9.38 µg/mL to 0.29 µg/mL) and CIP (4 µg/mL to 0.125 µg/mL) was analyzed by fitting into the bliss model (**Figure 5A**). A synergistic effect was observed across lower concentrations of QSL_Pd^5A^ and CIP and antagonistic effect was observed in higher concentrations of QSL_Pd5A (9.48 µg/mL) and CIP (4 µg/mL) with a bliss score of −104. Despite synergy being evident at 0.125 µg/mL with different QSL_Pd^5A^ concentrations, the most significant synergy score of 41.1 occurred when CIP was at 0.125 µg/mL and QSL_Pd^5A^ was at 4.69 µg/mL (**Figures 5B**). This resulted in a remarkable 64-fold reduction in the MIC of CIP against SA1199B. Thus, the bactericidal activity of CIP is significantly enhanced by QSL_Pd^5A^ compared to the individual effects of each drug on SA1199B.

### 3.7 Bactericidal kinetics assay

**Figure 6:**
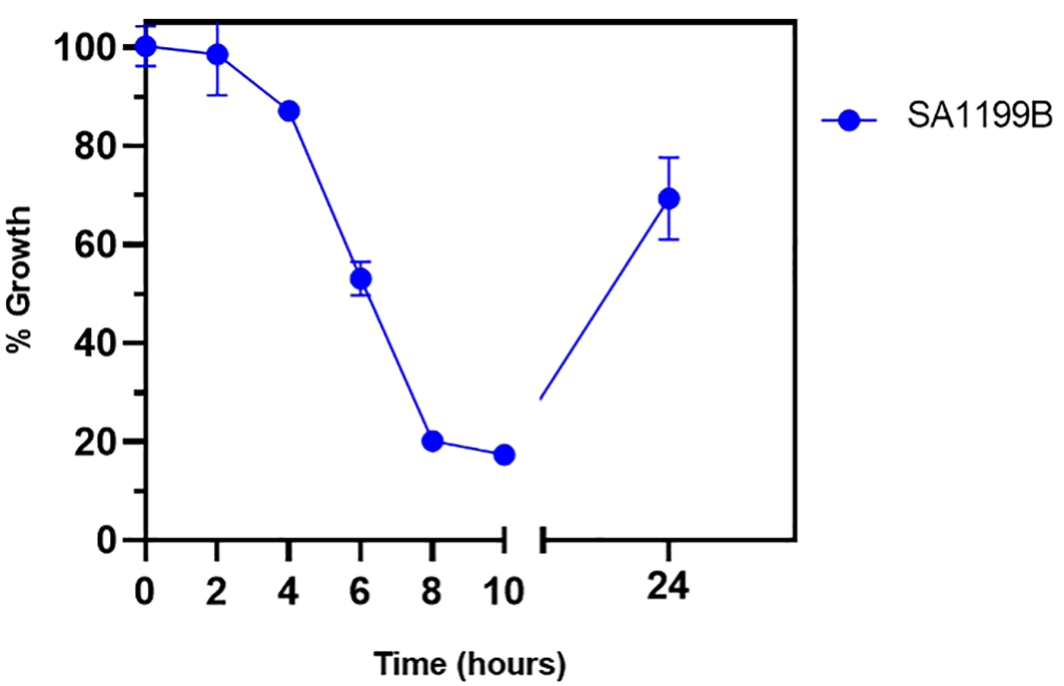
Bactericidal kinetics was evaluated for SA1199B after treatment with a constant concentration of ciprofloxacin at 0.125 µg/mL and QSL_Pd^5A^ at 4.69 µg/mL.

The time-kill kinetics assay was performed to understand the time-based effect of CIP along with QSL_Pd^5A^. SA1199B was exposed to CIP (at 0.125 µg/mL) along with QSL_Pd^5A^ (at 4.69 µg/mL) based on the results obtained from the combinatorial assay. A time-dependent decrease in the growth of the organism was observed. At the 8th hour, a significant inhibition in the growth (∼80%) was seen, and the effect tend to be retained for a short period (**Figure 6**).

### 3.8 Toxicity evaluation

**Figure 7:**
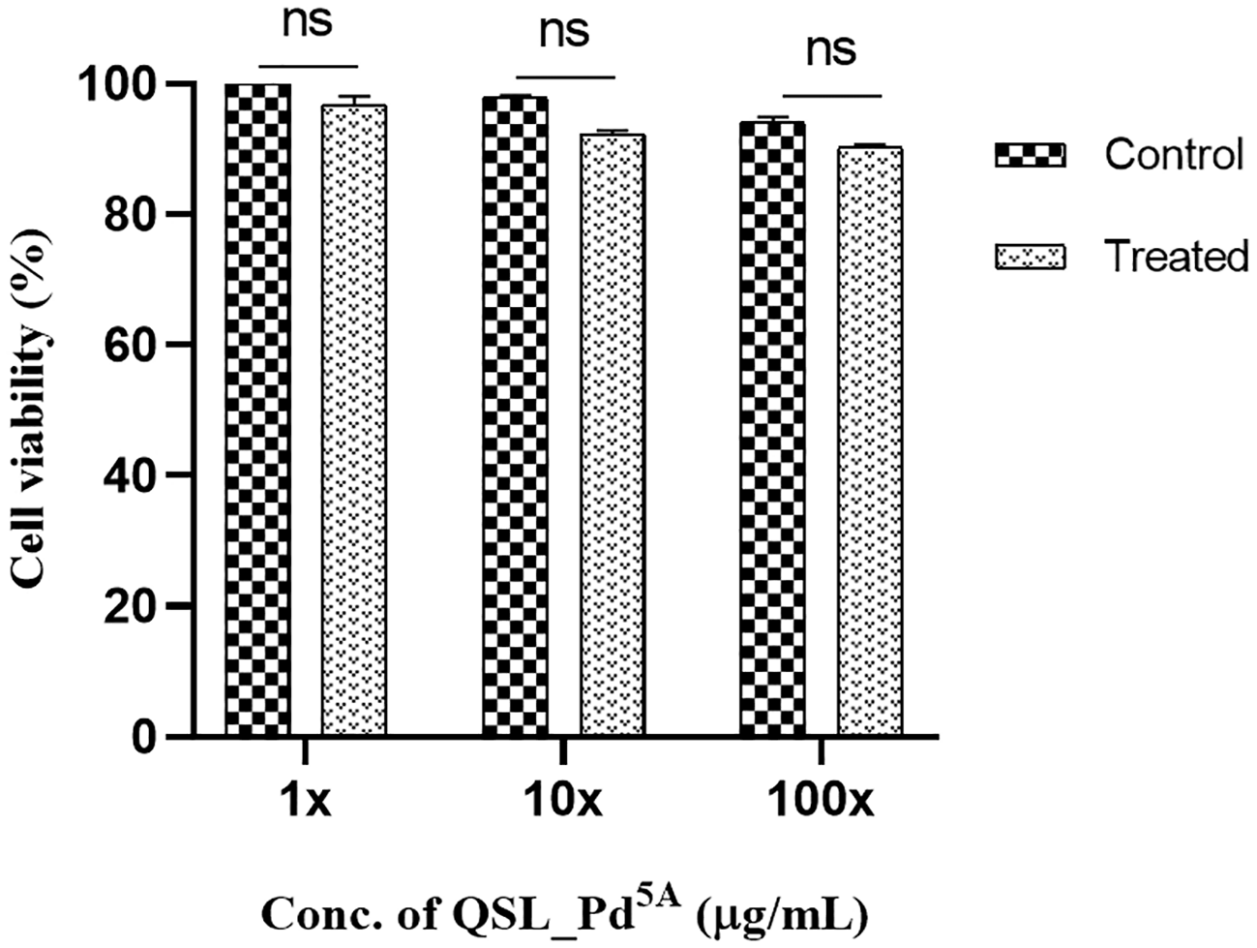
Cell viability of human kidney epithelial cells (NRK-62E) treated with QSL_Pd^5A^. No significant difference was observed between untreated and treated cells. The assay was done in triplicates, and the values were expressed as mean ± SD. NS denotes not significant (P > 0.05).

The MTT assay revealed dose-dependent toxicity in NRK-62E cells when exposed to the compound at its MIC concentration. However, the rate of cell death was insignificant. The percentage of cell viability remains similar to the control (untreated cells) even when cells were exposed to compound concentrations at 10× and 100× the MIC (**Figure 7**).

### 3.9 Docking studies

**Figure 8:**
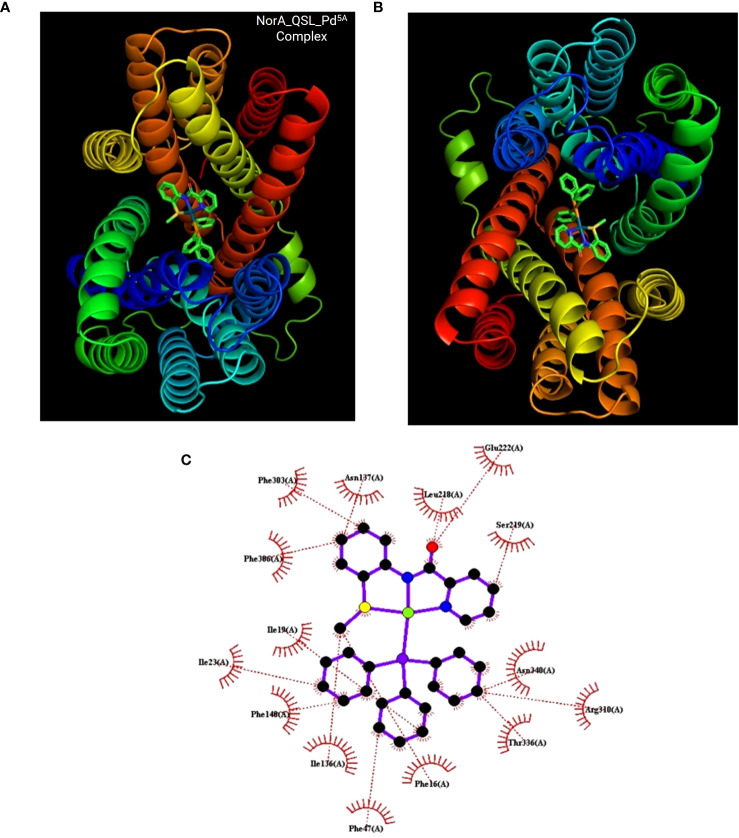
Docking results. **(A, B)** The NorA efflux pump (PDB ID:7LO7) bound to the compound QSL_Pd^5A^ at hydrophobic pocket was visualized using PyMol. **(C)** Interaction between them was visualization using LigPlus.

Docking was performed between NorA protein and QSL_Pd^5A^ and docking with ligand QSL_P^A^ and Verapamil (positive control) (**Figures 8A, B**). The binding energy score is given in **Table 3**. The binding energy of QSL_Pd^5A^ was greater than the positive control verapamil. The binding energy of QSL_Pd^5A^ was −8.41 kcal/mol, indicating higher stability and affinity of the palladium(II) complex toward the NorA protein. Thus, it proves that QSL_Pd^5A^ inhibits NorA EP. The interaction sites and amino acid residues at the binding sites are visualized using LigPlus (**Figure 8C**). There are more hydrophobic interactions between QSL_Pd^5A^ and NorA protein’s residues in the hydrophobic binding pocket like 16Phe, 19Ile, 23Ile, 47Phe, 140Phe, 222Glu, 306Phe, and 336Thr along with few pi-pi stacks, which are also observed (**Table S2**). **Table S3** depicts the amino acids and their position with their interaction type.

**Table 3:** Binding energy scores of QSL_Pd^5A^, QSL_P^A^, and verapamil.

Compound	Binding energy
QSL_Pd^5A^	−8.41 kcal/mol
QSL_P^A^	−5.90 kcal/mol
Verapamil	−6.26 kcal/mol

## 4 Discussion

Antibiotics, hailed as treatments for infections caused by microorganisms, are regrettably encountering resistance. The natural occurrence of antibiotic resistance happens when pathogens are exposed to antibiotics (Mancuso et al., 2021). Unfortunately, antimicrobial resistance is being exacerbated not only by the misuse of antibiotics but also by their inappropriate usage, such as wrong selection and intake of inadequate doses (Prestinaci et al., 2015). The multi-drug resistant is named super-bugs; one such microorganism is *Staphylococcus aureus. S. aureus* plays a dual role in the host by acting as a commensal and a pathogen. Typically, it colonizes the human body, including the skin and nasal passages, without rendering any harm. However, it can also cause infections ranging from mild to severe, depending on the strain and the site of infection (Torres Salazar et al., 2021). The waves of resistance acquired by *S. aureus* are alarming as they spread globally, leading to high mortality and morbidity rates (Chambers and DeLeo, 2009).

Hence, the need of the hour is an antibiotic with an entirely new core structure and no resistance mechanism developed against it. As mentioned in the introduction, the metal complex would help defend the antimicrobial resistance (AMR) due to its unique properties. Recently, Frei et al. determined the antibacterial and antifungal activity against *Enterococcus faecium*, *Staphylococcus aureus*, *Klebsiella pneumoniae*, *Acinetobacter baumannii*, *Pseudomonas aeruginosa*, and *Enterobacter spp.* (ESKAPE) pathogens and evaluated the cytotoxicity of 906 metal compounds available in the Community for Open Antimicrobial Drug Discovery database. Interestingly, the success rate was significantly higher (9.9%) than the organic molecules (0.87%) in the database. Among the metallofragments, ruthenium, silver, palladium, and iridium were found to be active and non-toxic to the cells. Thus, to break the double-edged sword, biofilm, and EP, various novel therapeutic approaches are emerging, like EPIs, to allow the accumulation of antibiotics inside the bacterial cells. In addition, some metal complexes, such as Ni(II), Cu(II), Mg(II), and Fe(III) complexes, have been identified to reduce MRSA biofilm formation. In addition, they function as EPIs, as reported by (Kincses et al., 2020a). These complexes exhibit higher lipophilicity than organic small-molecule inhibitors, enabling them to traverse the cell membrane more easily (Liang et al., 2021). Among these, palladium(II) complexes are less studied as potent EPI.

The current study reports the anti-biofilm and EP inhibition activity of palladium(II) complexes against *norA*-overexpressing strain SA1199B. The palladium(II) metal center exhibited a square planar geometry with three donor atoms from the tridentate ligand QSL_P^A^ or QSL_P^B^, and the fourth coordination comes from the substituted pyridyl or PPh_3_ or a ^–^C^-^CPh ligands. The ligands did not contribute to the antibacterial effect of the metal complex, which corroborates with the previously reported data (Abdulkarem, 2017). The tridentate ligand QSL_P^A^ and QSL_P^B^ differ in the S-donor atom being attached to the methyl group (S-Me) that constitutes “A” series and in the S-donor atom being attached to the phenyl group (S-Ph) that constitutes “B” series, respectively. The “A”-series compounds exhibited a high antibacterial effect at a lower concentration (MIC_50_ at 9.94 µg/mL to 24.7 µg/mL) than the “B”-series compounds, which would be primarily due to the higher hydrophobic nature of aliphatic R group (S-Me). Secondly, the less antibacterial effect exerted by the “B” series can be attributed to the steric hindrance exerted by the aromatic R group (S-Ph), leading to less interaction with the cell membrane and binding site.

Among various chromosomally encoded MDR EPs in *S. aureus*, NorA, a membrane transporter, explicitly extrudes hydrophilic fluoroquinolone antibiotics like ciprofloxacin and Norfloxacin, leading to antibiotic resistance (Faillace et al., 2021). The “A” series with low MIC_50_ was considered for the cartwheel assay and real-time EP inhibition. The accumulation of EtBr was visually and quantitatively confirmed. Although QSL_ Pd^2A^ had the maximum fluorescence intensity qualitatively, QSL_Pd^5A^ showed more potent inhibition of EP quantitatively. This demonstrated that the potential activity of palladium (II) metal complexes to inhibit EPs provided the rate of inhibition depends on the structure and functional groups in the complex.

The treatment of biofilm-mediated infections remains a clinical concern in the healthcare sector. The biofilm communities possess an upregulated EP that acts as a defense mechanism for the bacterial cells (Singh et al., 2017). Previous studies by Singh et al. and Sabatini et al. have reported the interrelationship between NorA EP inhibition and reduced biofilm formation in *S. aureus* (Sabatini et al., 2017; Singh et al., 2017). In accordance with these studies, QSL_Pd^5A^ retarded the formation of biofilm in a dose-dependent manner and was visualized using fluorescence imaging.

In the fight against AMR, there is not only a need for new antibiotics but also adjunct therapies with already available antibiotics (Shriram et al., 2018). To avoid the possibility of resistance, the EPI must not show antibacterial activity but act as a potent adjuvant to facilitate the activity of traditional antibiotics (Mahey et al., 2021). In this regard, the MIC of ciprofloxacin was reduced by 64-fold when in combination with QSL_Pd^5A^, consequently acting as potent armor to fight AMR. The synergism might be based on the differential mechanism due to administering an organic (CIP)-inorganic (Pd) cocktail (Eleftheriadou et al., 2021). Kincses et al. also reported the synergistic activity of various metal complexes [Zn(II), Cu, Mg, and Fe] in combination with CIP against *S. aureus* (Kincses et al., 2020a). In addition, various EPIs have also exhibited synergistic activity in combination with the fluoroquinolone class of antibiotics (Abd El-Baky et al., 2019; Faillace et al., 2021; Felicetti et al., 2022). To confirm the results obtained from the checkerboard assay and further determine the rate of bactericidal activity over time, a time-kill curve was plotted. A significant decrease in the growth was observed after 2 h. A similar effect was observed when Capsaicin and CIP were used in combination, the 8th hour observed the highest bactericidal activity and regrowth after the 12th hour (Kalia et al., 2012). Similarly, another study reported the bactericidal kinetics of boeravinone B with CIP, wherein the killing effect was maintained until the 12th hour (Singh et al., 2017). Still, in contrast, a bacteriostatic effect was observed after the 12th hour. This might be due to the lack of post-antibiotic effect by QSL_Pd^5A^ imparted on CIP.

To resolve the intermolecular interactions, *in silico* docking was performed for QSL_Pd^5A^, QSL_Pd^A^ ligand, and verapamil against NorA EP, among which QSL_Pd^5A^ had the highest binding affinity and stability at a binding energy of −8.41 kcal/mol with NorA. This result corroborates with the *in vitro* studies performed, demonstrating that the higher fluorescence in real-time efflux assay in treatment with QSL_Pd^5A^ was more than that in the verapamil. The decrease in the MIC of CIP, observed in the presence of QSL_Pd^5A^ against SA 1199B, may be linked to the prolonged presence of these substrates within bacterial cells. This extended presence is facilitated by the inhibition of the NorA EP, enabling CIP to reach their intended targets (dos Santos Barbosa et al., 2021). It is plausible that certain inhibitors exhibit a higher affinity for the hydrophobic pocket of EPs compared to efflux substrates. This heightened affinity allows these inhibitors to effectively block substrate binding, providing a rationale for the competitive action of QSL with the substrate (Aron and Opperman, 2018). Furthermore, much literature substantiates these findings, giving rise to the proposition that hydrophobicity plays a pivotal role in stabilizing the formation of complexes across diverse compound classes. These complexes demonstrate the ability to impair the functionality of EPs through hydrophobic and flexible H-bonding interactions (Sharom, 2008; Schweizer, 2012; Schindler and Kaatz, 2016).

### 5 Conclusion

The preliminary studies on QSL_Pd^5A^ demonstrated a substantial decrease in efflux activity and the effective prevention of biofilm formation. QSL_Pd5A’s inhibition of biofilm formation exposes planktonic cells to antibiotics at a remarkably low concentration. In addition, the compound impedes the extrusion of antibiotics, affirming a prolonged and sustained effect at a lower concentration of the conventional antibiotic. Importantly, the complex demonstrates no cytotoxicity, positioning it as a promising therapeutic molecule for topical applications. This opens avenues for further exploration of the compound through pharmacokinetic and pharmacodynamic studies. An in-depth *in vivo* investigation is required to assess the clinical efficacy of the compound as a pharmaceutical agent.

## Data availability statement

The original contributions presented in the study are included in the article/**Supplementary Material**. Further inquiries can be directed to the corresponding authors.

## Author contributions

RS: Conceptualization, Methodology, Writing – original draft. JT: Writing – original draft. SS: Writing – original draft. AJ: Writing – review & editing. AS: Conceptualization, Investigation, Methodology, Supervision, Writing – review & editing. DS: Conceptualization, Investigation, Writing – review & editing.

## References

[B1] Abd El-Baky R. M. Sandle T. John J. Abuo-Rahma G. E.-D. A. Hetta H. F. (2019). A novel mechanism of action of ketoconazole: inhibition of the NorA efflux pump system and biofilm formation in multidrug-resistant Staphylococcus aureus. Infection Drug Resistance 12, 1703–1718. doi: 10.2147/IDR.S201124 31354319 PMC6585162

[B2] Abdulkarem A. A. (2017). Synthesis and antibacterial studies of metal complexes of cu(II), ni(II) and co(II) with tetradentate ligand. J. Biophys. Chem. 08 (02), 13–21. doi: 10.4236/jbpc.2017.82002

[B3] Alav I. Sutton J. M. Rahman K. M. (2018). Role of bacterial efflux pumps in biofilm formation. J. Antimicrobial Chemotherapy 73 (8), 2003–2020. doi: 10.1093/jac/dky042 29506149

[B4] Álvarez-Martínez F. J. Barrajón-Catalán E. Micol V. (2020). Tackling antibiotic resistance with compounds of natural origin: A comprehensive review. Biomedicines 8 (10), 405. doi: 10.3390/biomedicines8100405 33050619 PMC7601869

[B5] Aron Z. Opperman T. J. (2018). The hydrophobic trap—the Achilles heel of RND efflux pumps. Res. Microbiol. 169 (7–8), 393–400. doi: 10.1016/j.resmic.2017.11.001 29146106 PMC5949246

[B6] Balamurugan P. Praveen Krishna V. Bharath D. Lavanya R. Vairaprakash P. Adline Princy S. (2017). Staphylococcus aureus quorum regulator sarA targeted compound, 2-[(Methylamino)methyl]phenol inhibits biofilm and down-regulates virulence genes. Front. Microbiol. 8. doi: 10.3389/fmicb.2017.01290 PMC5504099 28744275

[B7] Chambers H. F. DeLeo F. R. (2009). Waves of resistance: Staphylococcus aureus in the antibiotic era. Nat. Rev. Microbiol. 7 (9), 629–641. doi: 10.1038/nrmicro2200 19680247 PMC2871281

[B8] Chen C.-J. Huang Y.-C. (2014). New epidemiology of Staphylococcus aureus infection in Asia. Clin. Microbiol. Infection 20 (7), 605–623. doi: 10.1111/1469-0691.12705 24888414

[B9] Cheung G. Y. C. Bae J. S. Otto M. (2021). Pathogenicity and virulence of *Staphylococcus aureu* . Virulence 12 (1), 547–569. doi: 10.1080/21505594.2021.1878688 33522395 PMC7872022

[B10] Claudel M. Schwarte J. V. Fromm K. M. (2020). New antimicrobial strategies based on metal complexes. Chemistry 2 (4), 849–899. doi: 10.3390/chemistry2040056

[B11] Costa S. Junqueira E. Palma C. Viveiros M. Melo-Cristino J. Amaral L.. (2013a). Resistance to antimicrobials mediated by efflux pumps in staphylococcus aureus. Antibiotics 2 (1), 83–99. doi: 10.3390/antibiotics2010083 27029294 PMC4790300

[B12] Costa S. S. Viveiros M. Amaral L. Couto I. (2013b). Multidrug efflux pumps in staphylococcus aureus: an update. Open Microbiol. J. 7 (1), 59–71. doi: 10.2174/1874285801307010059 23569469 PMC3617543

[B13] de Sousa A. P. Gondim A. C. S. S. Sousa E. H. de França Lopes L. G. Teixeira E. H. Vasconcelos M. A.. (2020). Biphosphinic ruthenium complexes as the promising antimicrobial agents. New J. Chem. 44 (48), 21318–21325. doi: 10.1039/D0NJ03122D

[B14] dos Santos Barbosa C. R. Scherf J. R. de Freitas T. S. de Menezes I. R. A. Pereira R. L. S. dos Santos J. F. S.. (2021). Effect of Carvacrol and Thymol on NorA efflux pump inhibition in multidrug-resistant (MDR) Staphylococcus aureus strains. J. Bioenergetics Biomembranes 53 (4), 489–498. doi: 10.1007/s10863-021-09906-3 34159523

[B15] Egorova K. S. Ananikov V. P. (2017). Toxicity of metal compounds: knowledge and myths. Organometallics 36 (21), 4071–4090. doi: 10.1021/acs.organomet.7b00605

[B16] Eleftheriadou I. Giannousi K. Protonotariou E. Skoura L. Arsenakis M. Dendrinou-Samara C.. (2021). Cocktail of CuO, ZnO, or CuZn Nanoparticles and Antibiotics for Combating Multidrug-Resistant *Pseudomonas aeruginosa* via Efflux Pump Inhibition. ACS Appl. Nano Materials 4 (9), 9799–9810. doi: 10.1021/acsanm.1c02208

[B17] Faillace M. S. Alves Borges Leal A. L. Araújo de Oliveira Alcântara F. Ferreira J. H. L. de Siqueira-Júnior J. P. Sampaio Nogueira C. E.. (2021). Inhibition of the NorA efflux pump of S. aureus by (Z)-5-(4-Fluorobenzylidene)-Imidazolidines. Bioorganic Medicinal Chem. Lett. 31, 127670. doi: 10.1016/j.bmcl.2020.127670 33161124

[B18] Felicetti T. Cedraro N. Astolfi A. Cernicchi G. Mangiaterra G. Vaiasicca S.. (2022). New C-6 functionalized quinoline NorA inhibitors strongly synergize with ciprofloxacin against planktonic and biofilm growing resistant Staphylococcus aureus strains. Eur. J. Medicinal Chem. 241, 114656. doi: 10.1016/j.ejmech.2022.114656 35963131

[B19] Frei A. (2020). Metal complexes, an untapped source of antibiotic potential? Antibiotics 9 (2), 90. doi: 10.3390/antibiotics9020090 32085590 PMC7168053

[B20] Frei A. Verderosa A. D. Elliott A. G. Zuegg J. Blaskovich M. A. T. (2023). Metals to combat antimicrobial resistance. Nat. Rev. Chem. 7 (3), 202–224. doi: 10.1038/s41570-023-00463-4 37117903 PMC9907218

[B21] González-Ballesteros M. M. Mejía C. Ruiz-Azuara L. (2022). Metallodrugs: an approach against invasion and metastasis in cancer treatment. FEBS Open Bio 12 (5), 880–899. doi: 10.1002/2211-5463.13381 PMC9063434 35170871

[B22] Ikuta K. S. Swetschinski L. R. Robles Aguilar G. Sharara F. Mestrovic T. Gray A. P.. (2022). Global mortality associated with 33 bacterial pathogens in 2019: a systematic analysis for the Global Burden of Disease Study 2019. Lancet 400 (10369), 2221–2248. doi: 10.1016/S0140-6736(22)02185-7 36423648 PMC9763654

[B23] Kalia N. P. Mahajan P. Mehra R. Nargotra A. Sharma J. P. Koul S.. (2012). Capsaicin, a novel inhibitor of the NorA efflux pump, reduces the intracellular invasion of Staphylococcus aureus. J. Antimicrobial Chemotherapy 67 (10), 2401–2408. doi: 10.1093/jac/dks232 22807321

[B24] Kincses A. Szabó S. Rácz B. Szemerédi N. Watanabe G. Saijo R.. (2020a). Benzoxazole-based metal complexes to reverse multidrug resistance in bacteria. Antibiotics 9 (10), 649. doi: 10.3390/antibiotics9100649 32998217 PMC7600679

[B25] Liang J. Sun D. Yang Y. Li M. Li H. Chen L. (2021). Discovery of metal-based complexes as promising antimicrobial agents. Eur. J. Medicinal Chem. 224, 113696. doi: 10.1016/j.ejmech.2021.113696 34274828

[B26] Limbago B. (2001). M100-S11, Performance standards for antimicrobial susceptibility testing. Clin. Microbiol. Newslett. 23 (49), 88009-0. doi: 10.1016/S0196-4399(01)88009-0

[B27] Lloyd N. C. Morgan H. W. Nicholson B. K. Ronimus R. S. (2005). The composition of ehrlich’s salvarsan: resolution of a century-old debate. Angewandte Chemie Int. Edition 44 (6), 941–944. doi: 10.1002/anie.200461471 15624113

[B28] Mahey N. Tambat R. Chandal N. Verma D. K. Thakur K. G. Nandanwar H. (2021). Repurposing approved drugs as fluoroquinolone potentiators to overcome efflux pump resistance in staphylococcus aureus. Microbiol. Spectr. 9 (3). doi: 10.1128/Spectrum.00951-21 PMC8672906 34908453

[B29] Mancuso G. Midiri A. Gerace E. Biondo C. (2021). Bacterial antibiotic resistance: the most critical pathogens. Pathogens 10 (10), 1310. doi: 10.3390/pathogens10101310 34684258 PMC8541462

[B30] Martins M. McCusker M. P. Viveiros M. Couto I. Fanning S. Pagès J.-M.. (2013). A simple method for assessment of MDR bacteria for over-expressed efflux pumps. Open Microbiol. J. 7 (1), 72–82. doi: 10.2174/1874285801307010072 23589748 PMC3624690

[B31] Nasiri Sovari S. Zobi F. (2020). Recent studies on the antimicrobial activity of transition metal complexes of groups 6–12. Chemistry 2 (2), 418–452. doi: 10.3390/chemistry2020026

[B32] Piechota M. Kot B. Frankowska-Maciejewska A. Grużewska A. Woźniak-Kosek A. (2018). Biofilm formation by methicillin-resistant and methicillin-sensitive *staphylococcus aureus* strains from hospitalized patients in Poland. BioMed. Res. Int. 2018, 1–7. doi: 10.1155/2018/4657396 PMC6327255 30687745

[B33] Prestinaci F. Pezzotti P. Pantosti A. (2015). Antimicrobial resistance: a global multifaceted phenomenon. Pathog. Global Health 109 (7), 309–318. doi: 10.1179/2047773215Y.0000000030 PMC4768623 26343252

[B34] Rajaramon S. David H. Sajeevan A. Shanmugam K. Sriramulu H. Dandela R.. (2023). Multi-functional approach in the design of smart surfaces to mitigate bacterial infections: a review. Front. Cell. Infection Microbiol. 13. doi: 10.3389/fcimb.2023.1139026 PMC10242021 37287465

[B35] Sabatini S. Piccioni M. Felicetti T. De Marco S. Manfroni G. Pagiotti R.. (2017). Investigation on the effect of known potent S. aureus NorA efflux pump inhibitors on the staphylococcal biofilm formation. RSC Adv. 7 (59), 37007–37014. doi: 10.1039/C7RA03859C

[B36] Schindler B. D. Kaatz G. W. (2016). Multidrug efflux pumps of Gram-positive bacteria. Drug Resistance Updates 27, 1–13. doi: 10.1016/j.drup.2016.04.003 27449594

[B37] Schweizer H. P. (2012). Understanding efflux in Gram-negative bacteria: opportunities for drug discovery. Expert Opin. Drug Discovery 7 (7), 633–642. doi: 10.1517/17460441.2012.688949 22607346

[B38] Sharma A. Gupta V. Pathania R. (2019). Efflux pump inhibitors for bacterial pathogens: From bench to bedside. Indian J. Med. Res. 149 (2), 129. doi: 10.4103/ijmr.IJMR_2079_17 31219077 PMC6563736

[B39] Sharma B. Shukla S. Rattan R. Fatima M. Goel M. Bhat M.. (2022). Antimicrobial agents based on metal complexes: present situation and future prospects. Int. J. Biomaterials 2022, 1–21. doi: 10.1155/2022/6819080 PMC9754840 36531969

[B40] Sharom F. J. (2008). ABC multidrug transporters: structure, function and role in chemoresistance. Pharmacogenomics 9 (1), 105–127. doi: 10.2217/14622416.9.1.105 18154452

[B41] Shriram V. Khare T. Bhagwat R. Shukla R. Kumar V. (2018). Inhibiting bacterial drug efflux pumps via phyto-therapeutics to combat threatening antimicrobial resistance. Front. Microbiol. 9. doi: 10.3389/fmicb.2018.02990 PMC6295477 30619113

[B42] Singh S. Kalia N. P. Joshi P. Kumar A. Sharma P. R. Kumar A.. (2017). Boeravinone B, A novel dual inhibitor of norA bacterial efflux pump of staphylococcus aureus and human P-glycoprotein, reduces the biofilm formation and intracellular invasion of bacteria. Front. Microbiol. 8. doi: 10.3389/fmicb.2017.01868 PMC5632727 29046665

[B43] Sodhi R. K. (2019). Metal complexes in medicine: an overview and update from drug design perspective. Cancer Ther. Oncol. Int. J. 14 (2), 25–32. doi: 10.19080/CTOIJ.2019.14.555883

[B44] Thamilselvan G. Sarveswari H. B. Vasudevan S. Stanley A. Shanmugam K. Vairaprakash P.. (2021). Development of an antibiotic resistance breaker to resensitize drug-resistant staphylococcus aureus: in silico and *in vitro* approach. Front. Cell. Infection Microbiol. 11. doi: 10.3389/fcimb.2021.700198 PMC8415528 34485178

[B45] Torres Salazar B. O. Heilbronner S. Peschel A. Krismer B. (2021). Secondary metabolites governing microbiome interaction of staphylococcal pathogens and commensals. Microbial. Physiol. 31 (3), 198-216. 10.1159/000517082 34325424

[B46] Vasudevan S. Thamil Selvan G. Bhaskaran S. Hari N. Solomon A. P.. (2020). Reciprocal cooperation of type a procyanidin and nitrofurantoin against multi-drug resistant (mdr) upec: A ph-dependent study. Front. Cell. Infect. Microbiol. 10, 421. 32850505 10.3389/fcimb.2020.00421 PMC7431559

[B47] World Health Organization (WHO). (2017). Global priority list of antibiotic-resistant bacteria to guide research, discovery, and development of new antibiotics 2017, (Geneva: WHO Press), 1–7.

[B48] Zhang P. Sadler P. J. (2017). Redox-active metal complexes for anticancer therapy. Eur. J. Inorganic Chem. 2017 (12), 1541–1548. doi: 10.1002/ejic.201600908

