# Supplementary material for: Effect of palladium(II) complexes on NorA efflux pump inhibition and resensitization of fluoroquinolone-resistant Staphylococcus aureus: in vitro and in silico approach
Source: Front Cell Infect Microbiol. 2024 Jan 15;13:1340135. doi: 10.3389/fcimb.2023.1340135 (PMC10825952; doi:10.3389/fcimb.2023.1340135)

Supplementary Material

Effect of Palladium(II) Complexes on NorA Efflux Pump Inhibition and Resensitization of Fluoroquinolone Resistant *Staphylococcus aureus*: *In vitro* and *In silico* Approach

Rajaramon Shobana ^1†^, Jaffer Hussain Thahirunnisa ^1†^, Selvam Sivaprakash^2^, Arlin Jose Amali^2^, Adline princy Solomon ^1^* and Devarajan Suresh^2^*

† - These authors contributed equally to this work and share first authorship

***Correspondence:**

*****Prof. Adline Princy Solomon

[adlineprinzy@sastra.ac.in](mailto:adlineprinzy@sastra.ac.in)

*****Dr.D.Suresh

[suresh_d@scbt.sastra.edu](mailto:suresh_d@scbt.sastra.edu)


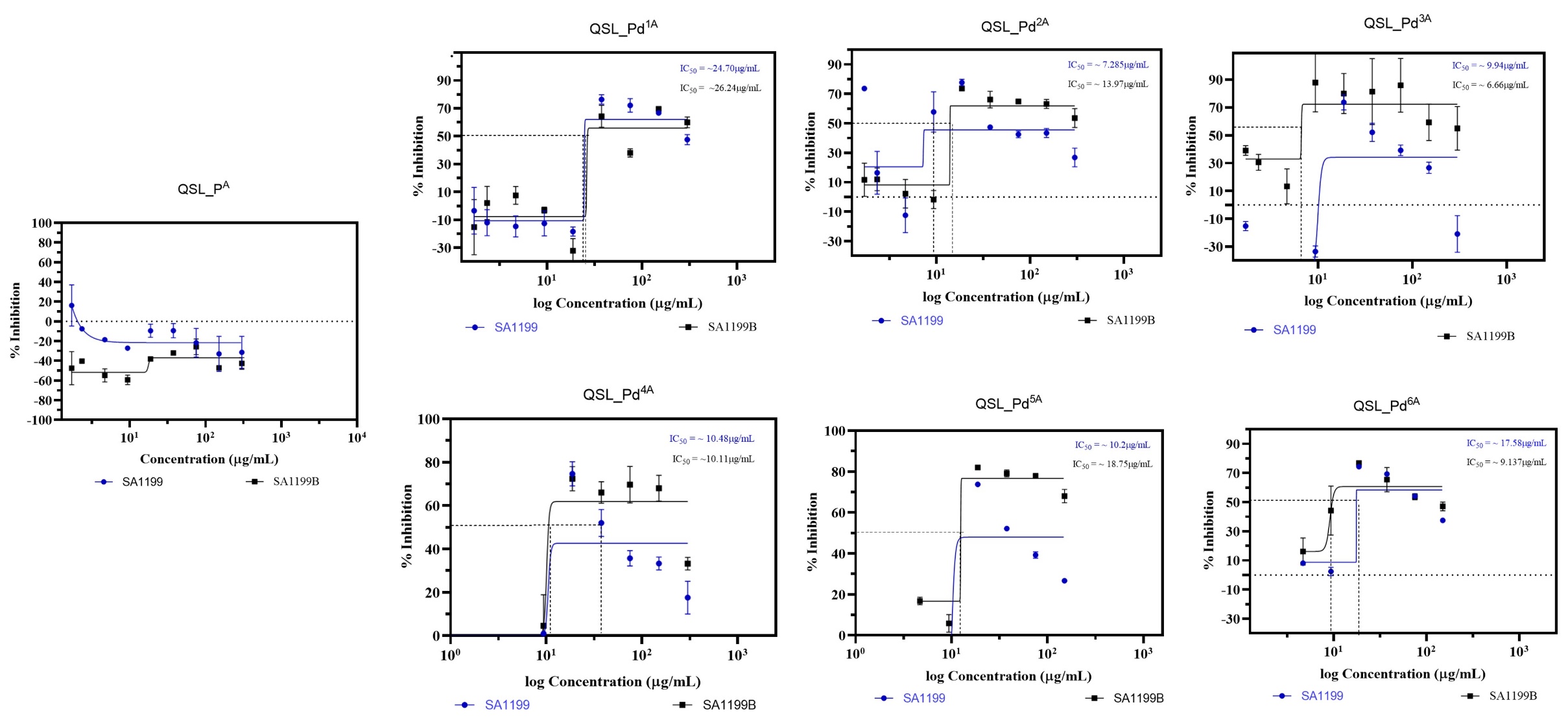
 **(a)**

**(b)**


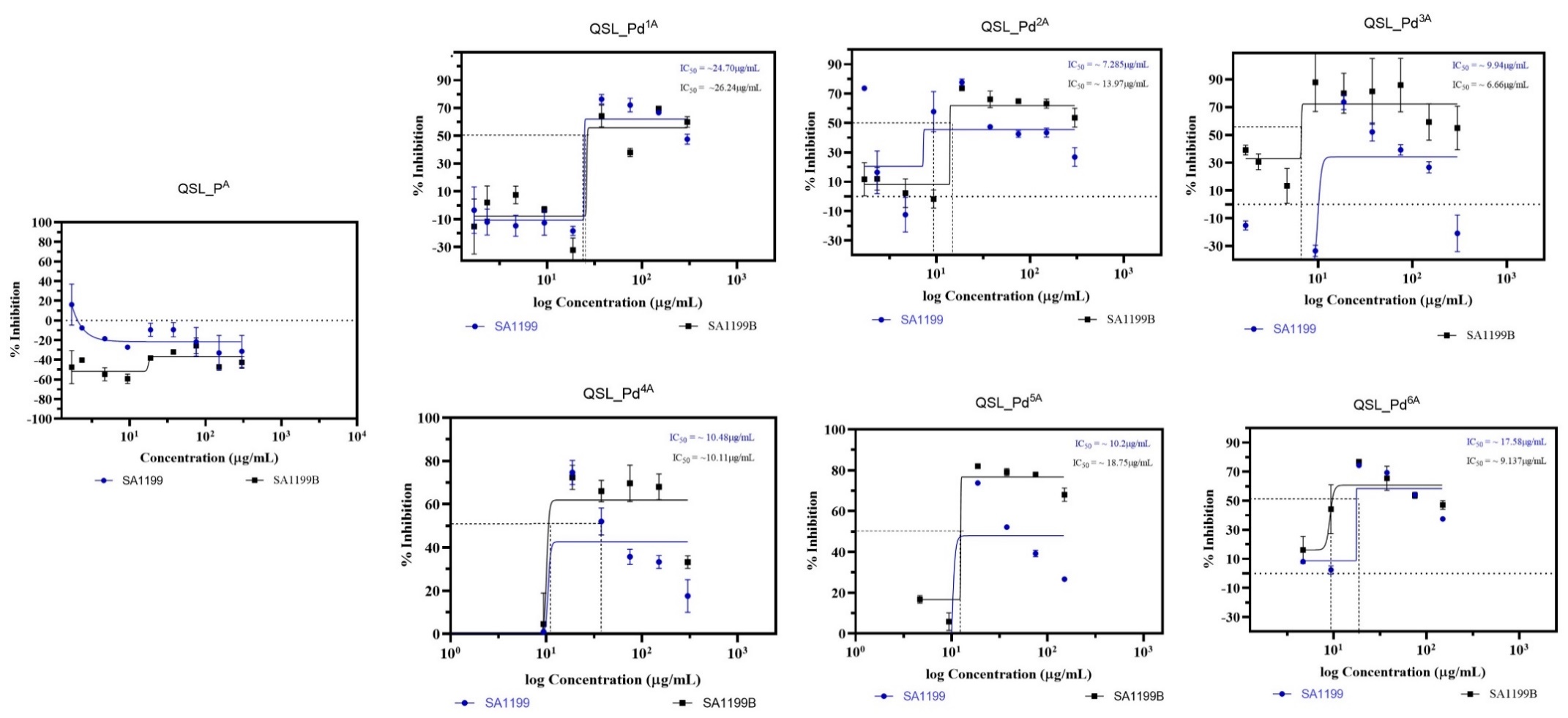


**Figure S1.** Dose-Response inhibition graph for “A”-series (**a)** QSL_P^A^ (ligand) **(b)** QSL_Pd^1A^- QSL_Pd^6A^ **(n=3).**

**(a)**

**
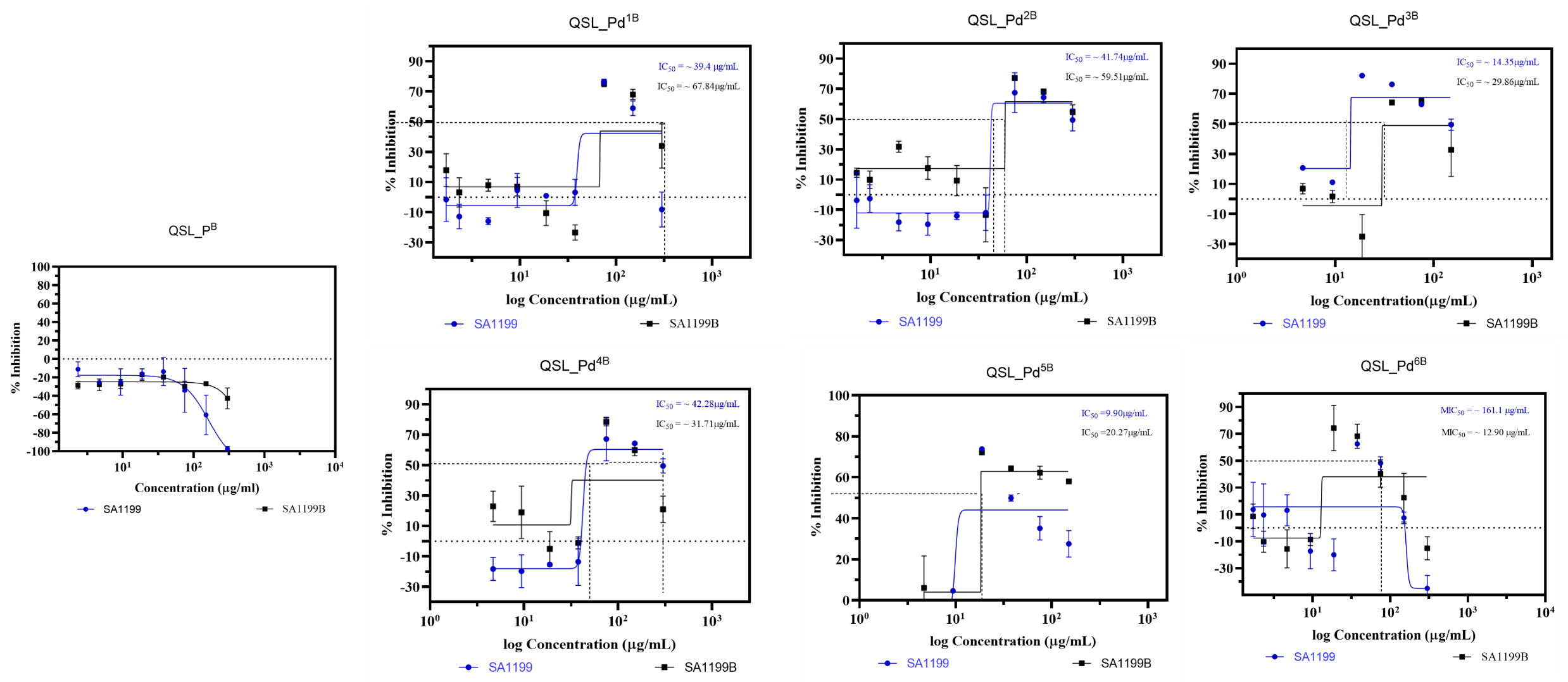
**

**(b)**

**
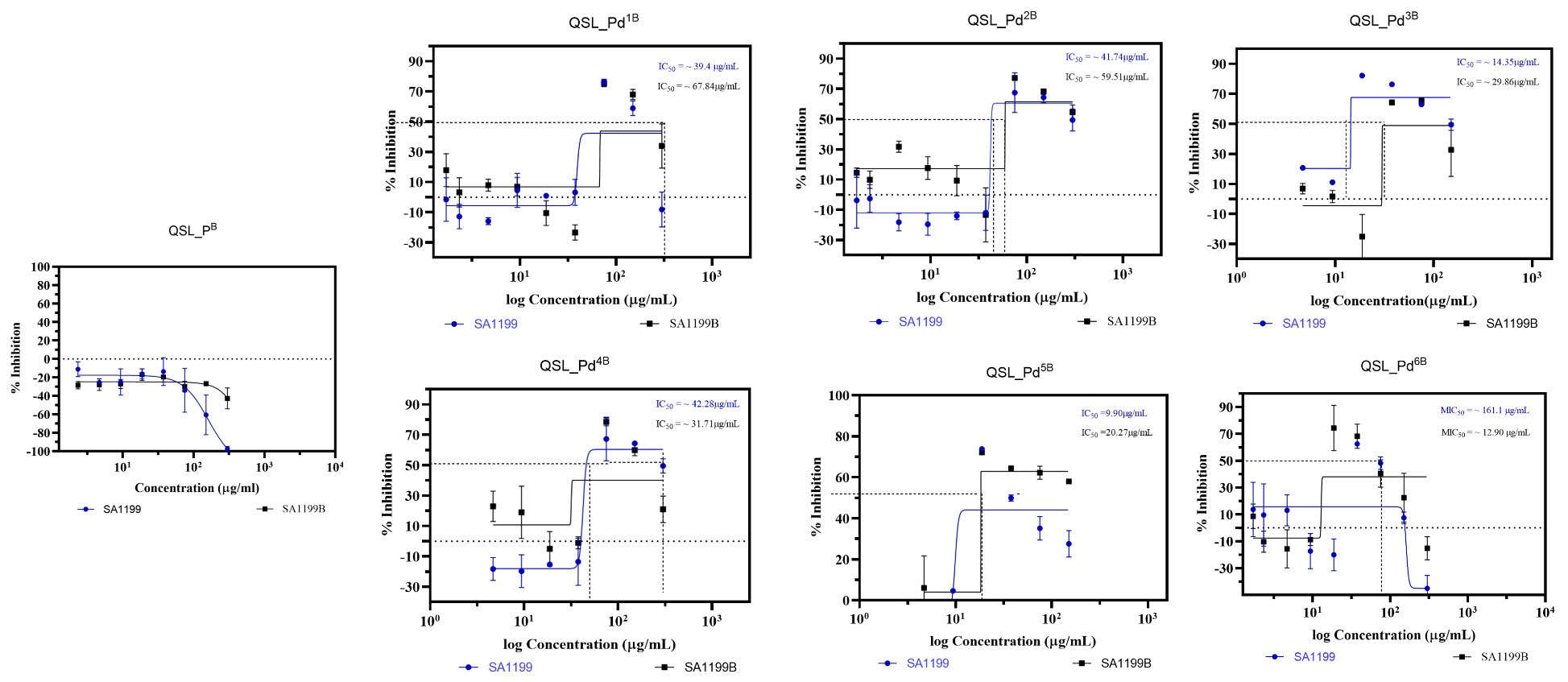
**

**Figure S2.** Dose-Response inhibition graph for “B”-series **a)** QSL_P^B^ (ligand) **b)** QSL_Pd^1B^- QSL_Pd^6^

**
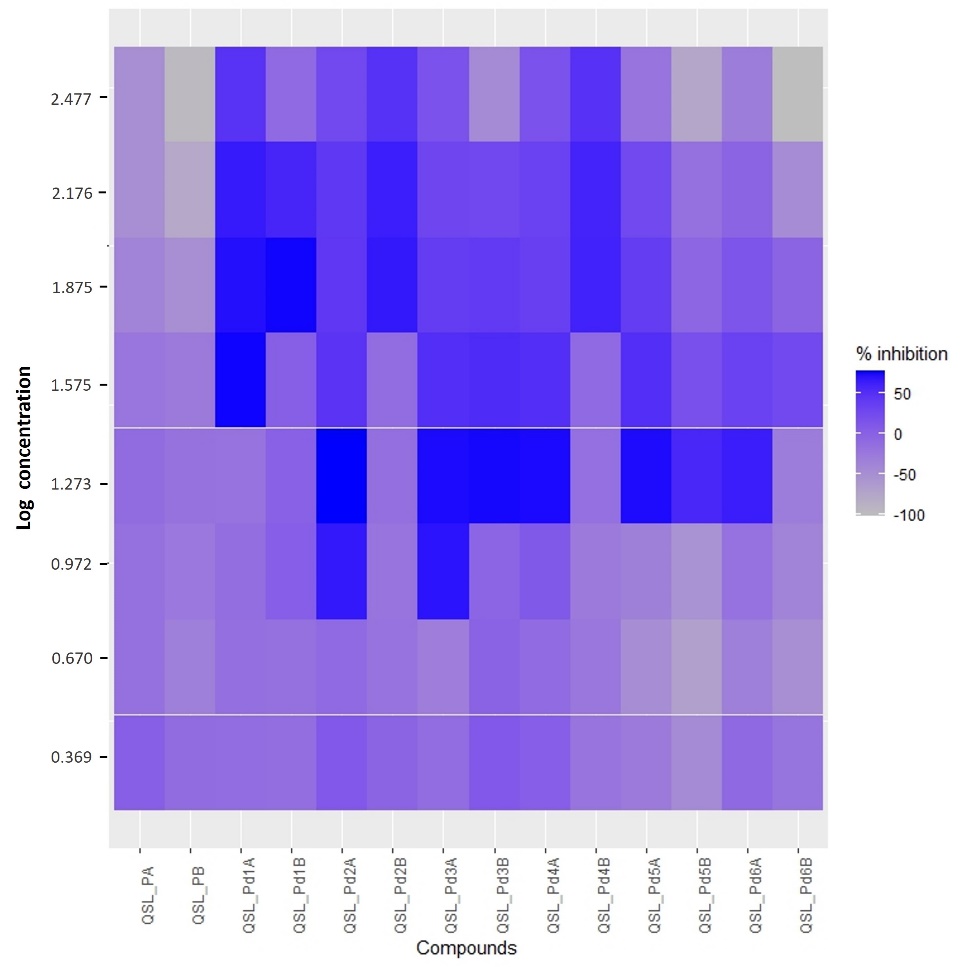

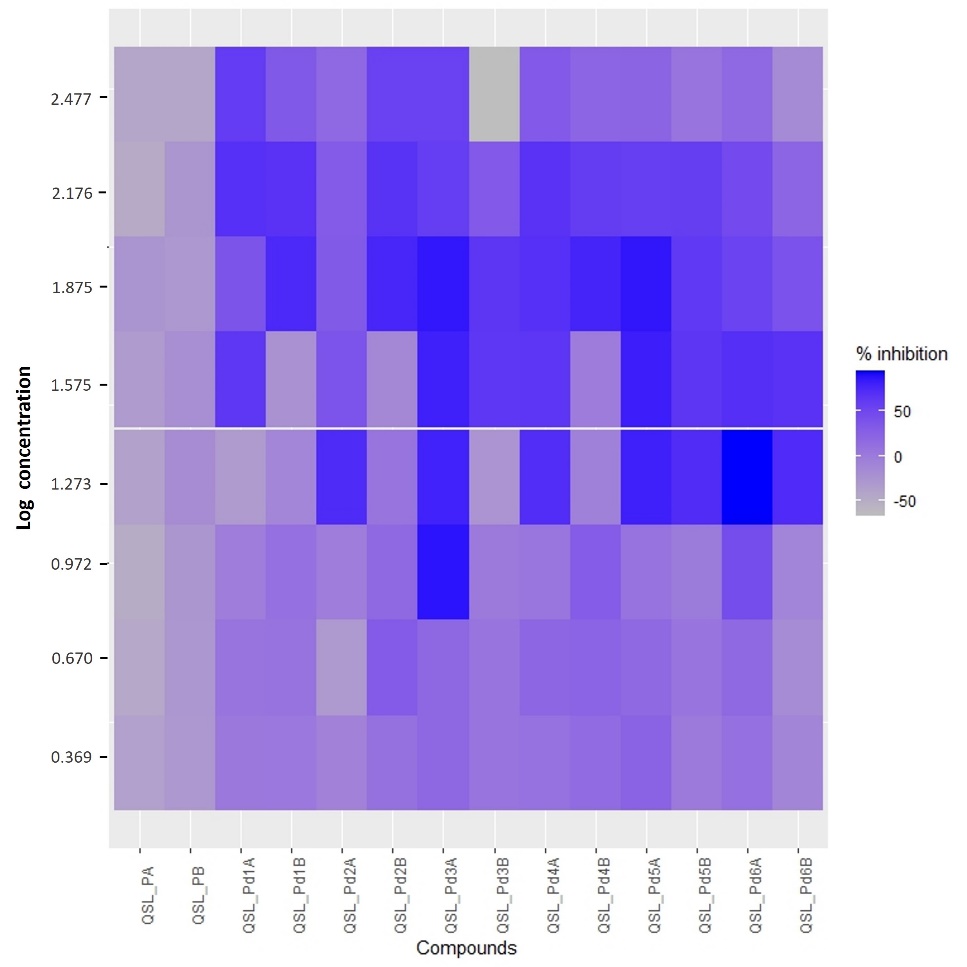
**

**Figure S3.** Heatmap depicting the % growth inhibition exerted by the evaluated palladium(II) metal complexes against Left: SA1199 and Right: SA1199B at varying concentrations. The “A”-series exhibited MIC_50_ at a lower concentration in comparison to the “B”-series. The color intensity directly correlates with the % inhibition.

**Table S1.** The fluorescence intensity emitted by EtBr under a UV trans illuminator was denoted with the symbol “+”( + -less intensity, ++ - high intensity, +++ - very high intensity).

|  | **SA1199 Nor A Inhibition** | **SA1199 B Nor A Inhibition** |
| --- | --- | --- |
| Control | - | - |
| QSL_Pd^A^ | - | - |
| QSL_Pd^1A^ | ++ | ++ |
| QSL_Pd^2A^ | +++ | +++ |
| QSL_Pd^3A^ | ++ | ++ |
| QSL_Pd^4A^ | + | + |
| QSL_Pd^5A^ | ++ | ++ |
| QSL_Pd^6A^ | + | + |

**Table S2.** Hydrophobic interactions between QSL_Pd^5A^ and NorA


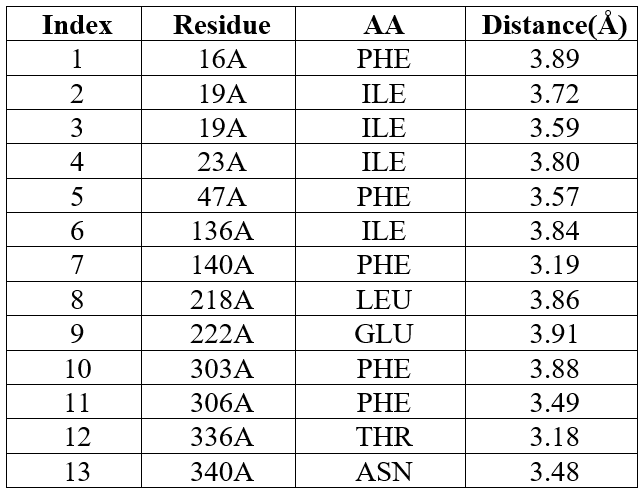


**Table S3.** π-Stacking between QSL_Pd^5A^ and NorA


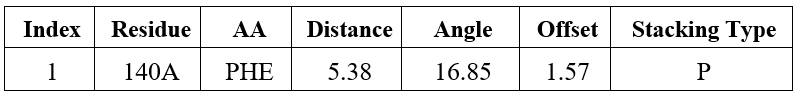

Supplement: Supplementary file 1 [file DataSheet_1.docx]
